# The Role of Endogenous Strigolactones and Their Interaction with ABA during the Infection Process of the Parasitic Weed *Phelipanche ramosa* in Tomato Plants

**DOI:** 10.3389/fpls.2017.00392

**Published:** 2017-03-24

**Authors:** Xi Cheng, Kristýna Floková, Harro Bouwmeester, Carolien Ruyter-Spira

**Affiliations:** ^1^Laboratory of Plant Physiology, Wageningen UniversityWageningen, Netherlands; ^2^Laboratory of Growth Regulators, Centre of the Region Haná for Biotechnological and Agricultural Research, Institute of Experimental Botany AS CR and Faculty of Science, Palacký UniversityOlomouc, Czechia

**Keywords:** root parasitic plant, strigolactone, abscisic acid, post-attachment resistance, plant architecture

## Abstract

The root parasitic plant species *Phelipanche ramosa*, branched broomrape, causes severe damage to economically important crops such as tomato. Its seed germination is triggered by host-derived signals upon which it invades the host root. In tomato, strigolactones (SLs) are the main germination stimulants for *P. ramosa*. Therefore, the development of low SL-producing lines may be an approach to combat the parasitic weed problem. However, since SLs are also a plant hormone controlling many aspects of plant development, SL deficiency may also have an effect on post-germination stages of the infection process, during the parasite-host interaction. In this study, we show that SL-deficient tomato plants (*Solanum lycopersicum; SlCCD8* RNAi lines), infected with pre-germinated *P. ramosa* seeds, display an increased infection level and faster development of the parasite, which suggests a positive role for SLs in the host defense against parasitic plant invasion. Furthermore, we show that SL-deficient tomato plants lose their characteristic SL-deficient phenotype during an infection with *P. ramosa* through a reduction in the number of internodes and the number and length of secondary branches. Infection with *P. ramosa* resulted in increased levels of abscisic acid (ABA) in the leaves and roots of both wild type and SL-deficient lines. Upon parasite infection, the level of the conjugate ABA-glucose ester (ABA-GE) also increased in leaves of both wild type and SL-deficient lines and in roots of one SL-deficient line. The uninfected SL-deficient lines had a higher leaf ABA-GE level than the wild type. Despite the high levels of ABA, stomatal aperture and water loss rate were not affected by parasite infection in the SL-deficient line, while in wild type tomato stomatal aperture and water loss increased upon infection. Future studies are needed to further underpin the role that SLs play in the interaction of hosts with parasitic plants and which other plant hormones interact with the SLs during this process.

## Introduction

During evolution, parasitic plants have evolved a mechanism to infect and rely on other plant species’ water and nutrients for their growth and survival. The root parasitic *Phelipanche ramosa* (*P. ramosa*), poses a severe threat to several economically important crops, particularly *Solanaceae* spp. (Parker, 2009). In tomato (*Solanum lycopersicum*), for example, an infection with this parasite leads to a large reduction in fruit biomass, mesocarp thickness, fruit color as well as changed contents of sugars and soluble solids in the fruits (Cagáň and Tóth, 2003; Longo et al., 2010).

The life cycle of *P. ramosa* consists of several different stages. Intriguingly, these parasites have evolved a mechanism ensuring that they only germinate within the hosts’ rhizosphere. This feature is very important since they cannot survive long after germination unless they reach their hosts’ root. Host-derived germination stimulants, such as strigolactones (SLs), have been described to be responsible for the induction of the germination of *P. ramosa* seeds (Bouwmeester et al., 2003). After seed germination, *P. ramosa* makes physical contact with its host by developing an attachment organ, a haustorium, which facilitates the establishment of a vascular connection between the parasite and its host. As the development of the vascular connection proceeds, a swollen organ, called a tubercle, is formed on the surface of the host root, enabling the accumulation of nutrients supporting further development of the parasite seedling. In a later stage, adventitious roots and apical shoot buds are formed at the base of the tubercle. Finally, the shoots of mature parasitic plants emerge above the soil (Xie et al., 2010; Cardoso et al., 2011).

Several hormones that are major players in signaling networks during other plant defense responses have been demonstrated to also play a role in the host–parasite interaction. Several reports have shown that genes involved in the jasmonic acid (JA) and/or ethylene pathways are induced in the roots of Arabidopsis (*Arabidopsis thaliana*), medicago (*Medicago truncatula*), lotus (*Lotus japonicus*) and tomato upon infection by *Orobanche* and *Phelipanche* spp. (Dos Santos et al., 2003a; Die et al., 2007; Dita et al., 2009; Hiraoka et al., 2009; Torres-Vera et al., 2016). In tomato and sunflower (*Helianthus annuus*), an infection with these parasites induced the expression of genes involved in the salicylic acid (SA) pathway (Letousey et al., 2007; Torres-Vera et al., 2016). In sunflower, a higher expression of these genes was found to be correlated with a more resistant phenotype (Letousey et al., 2007). In addition, application of methyl JA or methyl salicylate to Arabidopsis seedlings was able to evoke an almost full defense response during an infection with *Phelipanche aegyptiaca*, reducing attachment and tubercle formation by 90% (Bar-Nun and Mayer, 2008). However, this process is complicated by hormonal conjugations (Bar-Nun and Mayer, 2008).

Also abscisic acid (ABA) seems to be involved in the host–parasite interaction, as expression of ABA biosynthetic as well as ABA-responsive genes was induced in tomato upon *P. ramosa* infection (Torres-Vera et al., 2016). Proteomics showed that an abundance of ABA-responsive proteins is only detected in root extracts of an *Orobanche crenata*-resistant pea (*Pisum sativum*) cultivar (Angeles Castillejo et al., 2004), suggesting that ABA signaling is important for the plant’s defense against broomrape. ABA levels in sorghum and maize have also been reported to be elevated upon infection by the hemiparasite *Striga hermonthica* (Taylor et al., 1996; Frost et al., 1997). This seems not true for the association between the hemiparasite *Rhinanthus minor* and its host barley (*Hordeum vulgare*), in which ABA levels were not affected (Jiang et al., 2004, 2010).

The SLs are apocarotenoids like ABA, and are signaling molecules for parasitic plant seed germination and mycorrhizal symbiosis (Bouwmeester et al., 2003; Akiyama et al., 2005). Previously, it has been suggested that SLs and ABA influence each other’s levels, and it was shown that a SL-deficient tomato line (*SlCCD8* RNAi) had reduced levels of ABA when compared with its wild type (López-Ráez et al., 2010; Torres-Vera et al., 2016). Biosynthesis of the SLs is partially elucidated and was shown to be catalyzed by a number of enzymes, including DWARF 27 (D27), CAROTENOID CLEAVAGE DIOXYGENASE 7 (CCD7)/MORE AXILLARY GROWTH 3 (MAX3), CAROTENOID CLEAVAGE DIOXYGENASE 8 (CCD8)/MORE AXILLARY GROWTH 4 (MAX4), MORE AXILLARY GROWTH 1 (MAX1) and the recently identified LATERAL BRANCHING OXIDOREDUCTASE (LBO) (Booker et al., 2005; Lin et al., 2009; Alder et al., 2012; Kohlen et al., 2012; Abe et al., 2014; Zhang et al., 2014; Brewer et al., 2016). An F-box protein, MORE AXILLARY GROWTH 2 (MAX2)/DWARF3 (D3), an α/β-fold hydrolase DWARF 14 (D14) and DWARF 53 (D53), have been recognized as the main players in the SL signaling pathway (Arite et al., 2009; Mashiguchi et al., 2009; Nelson et al., 2011; Hamiaux et al., 2012; Jiang et al., 2013; Zhao et al., 2013; Chevalier et al., 2014; Kong et al., 2014).

Tomato lines with reduced SL production, such as the *SL-ORT1* mutant and *SlCCD8* RNAi lines, induce less *P. ramosa* germination, which results in reduced parasitic plant infection (Dor et al., 2011; Kohlen et al., 2012). Intriguingly, the expression of SL biosynthetic genes *MAX1*, *MAX3*, and *MAX4* is up-regulated in dodder pre-haustoria and haustoria (Ranjan et al., 2014), implying that SLs may play a role in the process of parasitism. Recently, SLs have also been reported to be involved in plant defense and stress responses (Bu et al., 2014; Ha et al., 2014; Torres-Vera et al., 2014; Liu J. et al., 2015). In addition, increased expression of SL biosynthetic genes *SlD27* and *SlCCD8* was observed in tomato roots after *P. ramosa* infection, suggesting activation of the SL biosynthetic pathway during the host–parasite interaction (Torres-Vera et al., 2016).

The aim of the present study is to investigate the role of SLs during the interaction between the host and parasite, other than in germination. We demonstrate a protective role for endogenous SLs after attachment of *P. ramosa* to tomato by comparing two independent SL-deficient *SlCCD8* RNAi lines with the corresponding wild type. We also observed that the parasite induced different phenotypic changes in the plant architecture of wild type and *SlCCD8* RNAi lines. To explore the relation between SLs and ABA in the regulation of the host response during this parasitic infection, we analyzed ABA levels, leaf water loss and stomatal features in the host. The role of SLs and the possible crosstalk with other hormones during the regulation of the defense response to parasitic plants is discussed.

## Materials and Methods

### Tomato Materials and Plant Growth

In this study, tomato wild type (cv. Craigella) and *SlCCD8* RNAi lines (L04, L09), which have been described in a previous study (Kohlen et al., 2012), were used. Tomato seeds were germinated on moistened filter paper at 25°C for 4 days in darkness. Germinated tomato seeds were selected and grown in moistened vermiculite, for 2 weeks for the rhizotron assay as described below, and for 3 weeks for the soil assay, under 12 h:12 h L: D photoperiod at 21°C in a growth chamber.

### *Phelipanche ramosa* Infection in a Rhizotron System

A rhizotron system was adapted from previous studies on rice-*Striga* interactions (Gurney et al., 2006; Cissoko et al., 2011). The rhizotron was prepared by cutting a hole at one side of a 14.5 cm-diameter round Petri dish. The Petri dish was filled with a 1.5 cm thick piece of round rockwool, a round glass-fiber filter disk (Whatman GF/A paper), and finally a nylon mesh on top. The rhizotron system was moistened with sterilized ½-strength Hoagland nutrient solution. Sterile seedlings were then moved to the rhizotron system by fitting the plant in the hole of the Petri dish. The leaves and shoots of the seedlings were kept outside while the roots were carefully separated and organized on top of the nylon mesh using forceps. The rhizotron system was placed in a vertical position at 21°C, 60% RH, 100 μmol m^-2^ s^-1^ light intensity, in a 12 h:12 h L: D photoperiod and plants grown for another 2 weeks.

At the same time, *P. ramosa* seeds were sterilized with a 2% bleach solution and five drops of Tween20 for 5 min, and then washed with sterile demineralized water. Sterile *P. ramosa* seeds were dried and applied to a 5 cm-diameter glass-fiber filter paper (Whatman GF/A paper), which was pre-wetted with 0.8 ml sterile demineralized water and placed in 9 cm-diameter Petri dishes with a pre-wetted 1 cm-wide ring of filter paper to maintain moisture. The Petri dishes were sealed with parafilm and then kept in the dark in a growth chamber at 20°C for a 12 days pre-conditioning period. Pre-conditioned seeds were then dried and treated with 0.8 ml of a 3.3 × 10^-3^μM GR24 (synthetic strigolactone analog) solution for 24 h in the dark at 25°C. GR24 treatment triggered the germination of *P. ramosa*. After 24 h, GR24 was washed off with sterile demineralized water. The pre-germinated *P. ramosa* seeds were then spread along the roots of the 2-week old tomato seedlings in the rhizotron system using a sterile paint brush. The rhizotron Petri dishes were sealed with tape and covered with aluminum foil. The plates were then placed vertically again and the plants were grown under the same conditions as described above for another 4 weeks. Rhizotron Petri dishes were randomly placed in trays and their positions were randomized again every 3 days. Photos of *P. ramosa*-infested roots in the rhizotron were taken with a Canon digital camera EOS 60D DSLR (with EF-S 18–135 mm IS Lens) 15 and 32 days post-inoculation (dpi).

### *Phelipanche ramosa* Infection Assay in Soil and Host Phenotype Analysis

Seeds of tomato wild type (cv. Craigella) and *SlCCD8* RNAi line L09 (five replicates) were germinated on moistened filter paper at 25°C for 4 days in darkness. Germinated seeds were moved onto moistened vermiculite for 2 weeks using a 12 h:12 h L: D photoperiod at 21°C, 60% RH, 100 μmol m^-2^ s^-1^ light intensity in a growth chamber. Young tomato seedlings were carefully pulled out of the vermiculite substrate and their roots were cleaned with water. Pre-germinated *P. ramosa* seeds were applied to each tomato roots by using a paint brush (15 mg *P. ramosa* seeds per tomato plant). Tomato seedlings were then planted in a mixture of soil: vermiculite: sand (1:1:1) and grown at 25°C, 60% RH, 150 μmol m^-2^ s^-1^ light intensity and 16 h:8 h L: D photoperiod in the greenhouse. After 9 weeks, the number of above-ground emerging *P. ramosa* seedlings was counted. The branch number (primary and secondary branches) and internode number of *P. ramosa*-infected and uninfected tomato plants (wild type and the line L09, L04) were counted. The length of tomato branches (primary and secondary branches) and each internode were also measured. Subsequently, the soil was washed off the tomato roots. The parasitic tubercles and shoots were carefully detached from the tomato roots. The weight of stem, leaves, shoots, branches, and roots of *P. ramosa*-infected and uninfected wild type and L09 tomato plants, and the total weight of the parasitic plant biomass were measured.

### Measurement of Stomatal Aperture and Conductance

Two leaves, of approximately similar age and not covered by other leaves, were collected from three plants for wild type and L09 with/without *P. ramosa* (five biological replicates). To make stomatal imprints, vinylpolysiloxane dental resin was applied to the abaxial side of the leaf at midday by using a dispensing gun (Dispenser D2, Zhermack SpA, Italy) and removed after drying. The resin imprints were covered with transparent nail polish which was then peeled off after drying, giving a mirror image of the resin imprint. Photos of stomata were then taken of the imprints using a digital camera Nikon DIGITAL SIGHT DS-Fi1 (Nikon Instruments, Inc.) and acquired with Nikon NIS-Elements software. Ten photos per leaf imprint were subjected to image analysis using the software package ImageJ. Stomatal aperture was calculated as the ratio of stomatal length to width. Stomatal conductance was measured directly in leaves of *P. ramosa*-infected and uninfected wild type and L09 plants using a leaf porometer (Decagon Devices, Inc.) in the afternoon between 14:00 and 17:00 h.

### Leaf Dehydration Assay

Four full-grown leaves with similar age from the top of the plant (without coverage by other leaves) were detached from seedlings of wild type and *SlCCD8* RNAi line L09 with or without *P. ramosa* infection (five biological replicates) from the soil infection experiment (8 weeks after infection). Collected leaves were placed in open Petri dishes on a bench in the growth chamber (at 21°C, 60% RH, 100 μmol m^-2^ s^-1^ light intensity). Leaf weights were periodically measured at the indicated time points (0, 15, 30, 60, 90 min). Rate of leaf water loss was calculated as leaf weight loss divided by time.

### ABA Measurements

Young leaves and roots were collected from wild type and L09 seedlings with or without *P. ramosa* infection (five biological replicates, two technical replicates) from the soil infection experiment. ABA levels were measured in these samples by multiple reaction monitoring (MRM) using ultra-performance liquid chromatography coupled to tandem mass spectrometry (UPLC–MS/MS) using a published protocol (Floková et al., 2014).

### Statistical Analysis

Data are presented as mean ± standard error of the mean (SEM). Statistical analysis was performed using Student’s *t*-test (two-tailed) or analysis of variance (ANOVA) (GraphPad Prism version 7.00 for Windows). Differences between individual means were tested for significance using the *post hoc* Tukey’s multiple comparison test. Percentages of differentiated and undifferentiated tubercles and total tubercle percentage from the rhizotron studies were transformed using arcsine square root transformation of the raw data prior to statistical analysis.

## Results

### Endogenous SLs Inhibit Parasitism

**FIGURE 1:**
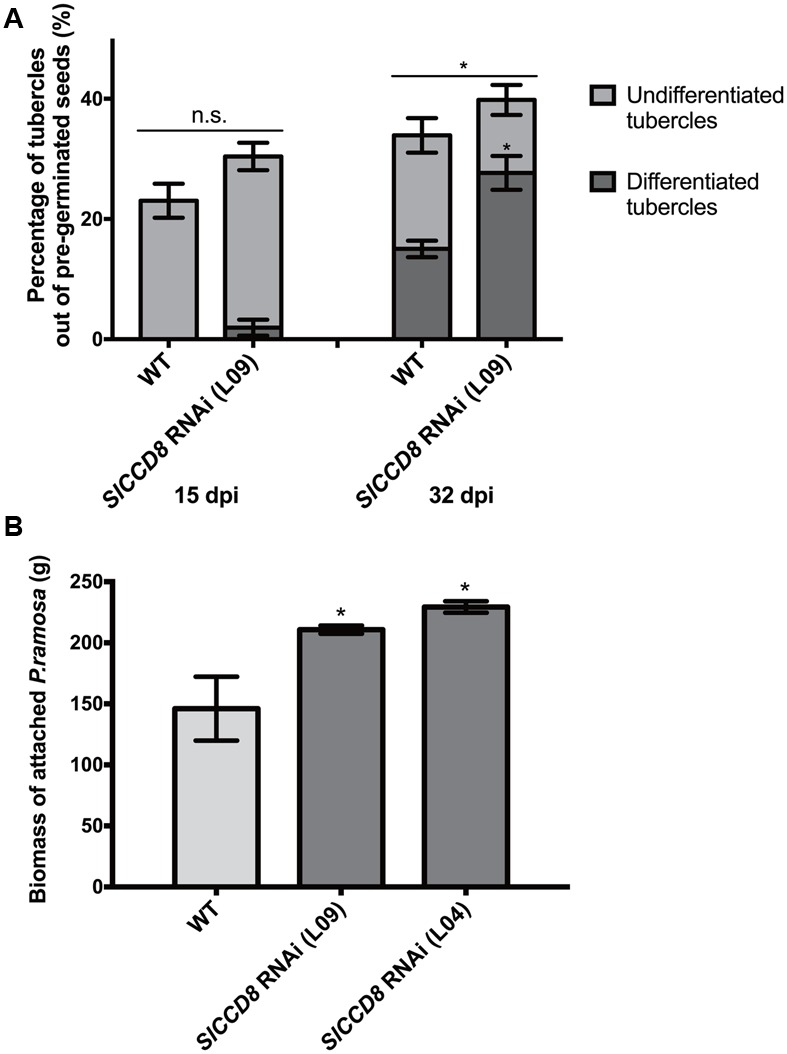
**Tomato strigolactone biosynthesis knock-down, *SlCCD8* RNAi lines, are more susceptible to *Phelipanche ramosa* infection than wild type. (A)** Percentage of undifferentiated (in light gray color) and differentiated tubercles (in dark gray color) (out of the total number of pre-germinated *P. ramosa* seeds applied around the host roots) that developed on wild type (WT) and *SlCCD8* RNAi line L09 at 15 dpi (days post-infection) and 32 dpi in the rhizotron assay. Original data was subjected to arcsine root transformation before statistical analysis. **(B)** Total fresh weight (biomass) of attached parasites on tomato seedlings of WT and two *SlCCD8* RNAi lines (L09 and L04) in the soil assay. Data represent the means of nine **(A)** or five **(B)** independent replicates ± standard error (SE). Asterisks (^∗^) indicate statistically significant differences between WT and *SlCCD8* RNAi lines (L09 and L04), respectively, according to Student’s *t*-test. ^∗^*P* < 0.05.

To assess the role of endogenous SLs on parasitic plant attachment in tomato, we studied the development and final number of *P. ramosa* tubercles using a rhizotron assay. In this assay, we compared the susceptibility of SL-deficient tomato *SlCCD8* RNAi line L09 with its wild type. Because we were specifically interested in differences in parasite attachment levels that were not the result of differences in *P. ramosa* seed germination, the parasitic plant seeds were pre-germinated, using the synthetic strigolactone analog GR24, before they were applied to the tomato roots. A higher overall level of parasitic plant infection was observed in L09 seedlings when compared with its wild type at 32 dpi (**Figure 1A**, *P* < 0.05). In addition to this, the percentage of differentiated tubercles (with adventitious roots) was also higher in the SL-deficient line L09 when compared to its wild type while the percentage of undifferentiated tubercles (without adventitious roots) was similar between wild type and L09 (**Figure 1A**, *P* < 0.05), suggestive of a faster development of the parasite on L09.

In addition to this semi *in vitro* assay, a soil infection assay was conducted using two independent tomato *SlCCD8* RNAi lines (L09 and L04) and the corresponding wild type. Also, in this experiment, the *P. ramosa* seeds were pre-germinated with GR24. Fresh weight of the attached parasites was measured after 9 weeks and was shown to be higher in both *SlCCD8* RNAi tomato lines compared with their wild type (**Figure 1B**). The combined results from both assays show that SL-deficient tomato plants are more susceptible to (pre-germinated) *P. ramosa* infection and sustain a faster development of the parasite.

### *P. ramosa* Infection Affects Tomato Shoot Architecture Differently in WT and *SlCCD8* RNAi Lines

**FIGURE 2:**
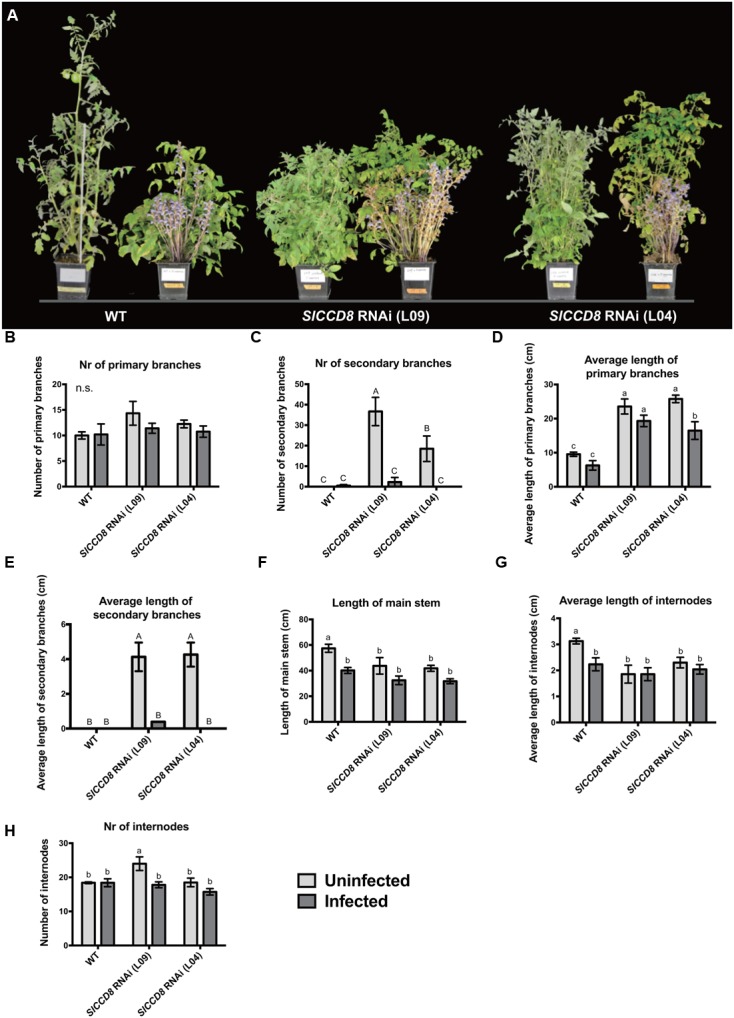
**Shoot architecture of wild type (WT) and *SlCCD8* RNAi lines (L09 and L04) uninfected and infected with the root parasitic *P. ramosa*. (A)** Picture of the 12-week-old wild type and *SlCCD8* tomato RNAi lines, uninfected (left plant) and infected (right plant) with *P. ramosa*. **(B)** Number of primary branches; **(C)** number of secondary branches; **(D)** length of primary branches (cm); **(E)** length of secondary branches (cm); **(F)** length of main stem (cm); **(G)** average length of internode (cm); **(H)** number of internodes. Data represent the average of five independent replicates ± standard error (SE). Letters (a, b, c and A, B, C) indicate statistically significant differences according to two-way ANOVA (treatment and line as factors) and Tukey’s multiple comparisons tests (a, b, c; *P* < 0.05; A, B, C; *P* < 0.001); n.s.: no statistical significant differences for any of the comparisons in the respective graph.

To further explore the possible role of SLs in the host response to an infection with parasitic plants, we studied the effect of an infection with *P. ramosa* on the growth and plant architecture of the wild type and *SlCCD8* RNAi tomato lines. Without infection, *SlCCD8* RNAi plants (L09 and L04) displayed a more compact phenotype than the corresponding wild type, resulting from an increased number of branches and reduced plant height (**Figure 2A**). When the plants were infected with *P. ramosa* (**Figure 2A**), the shoot architecture of wild type plants became more compact due to a reduction in plant height (**Figure 2A**), while shoot branching in the *SlCCD8* RNAi lines was reduced with no obvious changes in plant height (**Figure 2A**).

To further investigate the effect of a *P. ramosa* infection on plant architecture of wild type and *SlCCD8* RNAi lines, parameters for shoot branching and stem growth were quantified (**Figure 2**). Compared to control conditions, the number and length of primary branches of wild type and *SlCCD8* RNAi lines remained unchanged during the infection, with the exception of L04 that displayed a reduction in the length of its primary branches (**Figures 2B,D**). However, the secondary branches of both *SlCCD8* RNAi lines displayed a remarkable reduction in both number and length during the infection with *P. ramosa* (**Figures 2C,E**, *P* < 0.001). This explains the less compact appearance of the infected *SlCCD8* RNAi lines observed in the pot experiment shown in **Figure 2A**. In a second pot experiment during which the *P. ramosa* infection was more severe, the same trend was observed while now also the number of primary branches of the *SlCCD8* RNAi lines was reduced (data not shown). The *P. ramosa* also caused a large reduction in stem length, but only in wild type plants (**Figure 2F**, *P* < 0.05). This was mainly caused by a reduction in the internode length rather than a reduction in the number of internodes (**Figures 2G,H**). This is consistent with the more compact and dwarf-like appearance of wild type plants upon infection with *P. ramosa* (**Figure 2A**).

**FIGURE 3:**
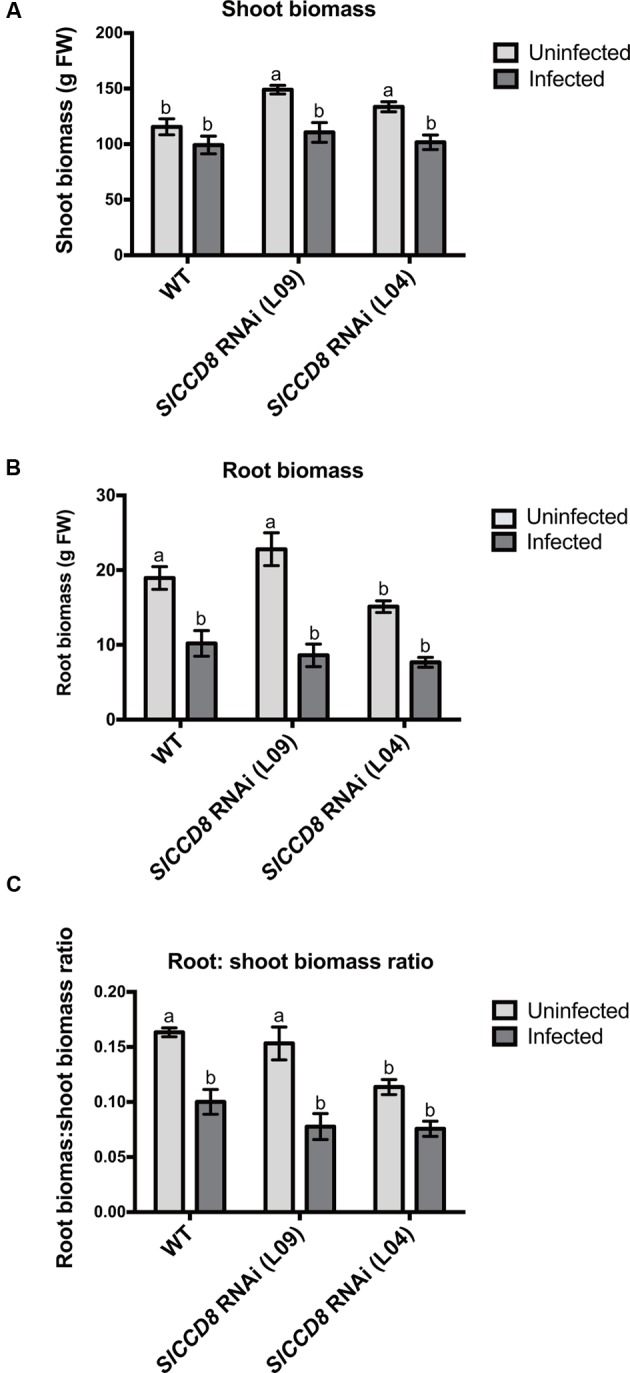
**Shoot biomass (A)**, root biomass **(B)** and root: shoot biomass ratio **(C)** of wild type (WT) and *SlCCD8* RNAi lines (L09 and L04) uninfected and infected with the root parasitic plant *P. ramosa*. Data represent the means of five independent replicates ± standard error (SE). Letters (a, b) indicate statistically significant differences according to two-way ANOVA (treatment and line as factors) and Tukey’s multiple comparisons test (*P* < 0.05).

In addition, shoot and root biomass (fresh weight) were measured in uninfected and infected wild type and the two SL-deficient lines (**Figure 3**). The *P. ramosa* infection significantly reduced root biomass of wild type plants (**Figure 3B**, *P* < 0.05), while shoot biomass of wild type remained unaffected (**Figure 3A**). However, the *P. ramosa* infection remarkably reduced both root and shoot biomass in both RNAi lines (although the reduction in root biomass of L04 was on the border of significance; adjusted *P*-value = 0.069) (**Figures 3A,B**). These results show that *P. ramosa* infection in wild type only reduces root biomass, while in the *SlCCD8* RNAi lines both root and shoot biomass are decreased. Upon infection, both wild type and L09 displayed a reduced root-to-shoot biomass ratio (**Figure 3C**, *P* < 0.05), implying that the negative effect of the infection on host root biomass is larger than the effect on shoot biomass.

In conclusion, *SlCCD8* RNAi lines are more susceptible to an infection with *P. ramosa*, and show a reduction in biomass in both roots and shoots. The reduction in shoot biomass in the *SlCCD8* RNAi lines is mainly caused by a reduction in the number and length of secondary shoot branches.

### *P. ramosa* Infection Affects ABA Levels, Stomata and Leaf Water Loss

**FIGURE 4:**
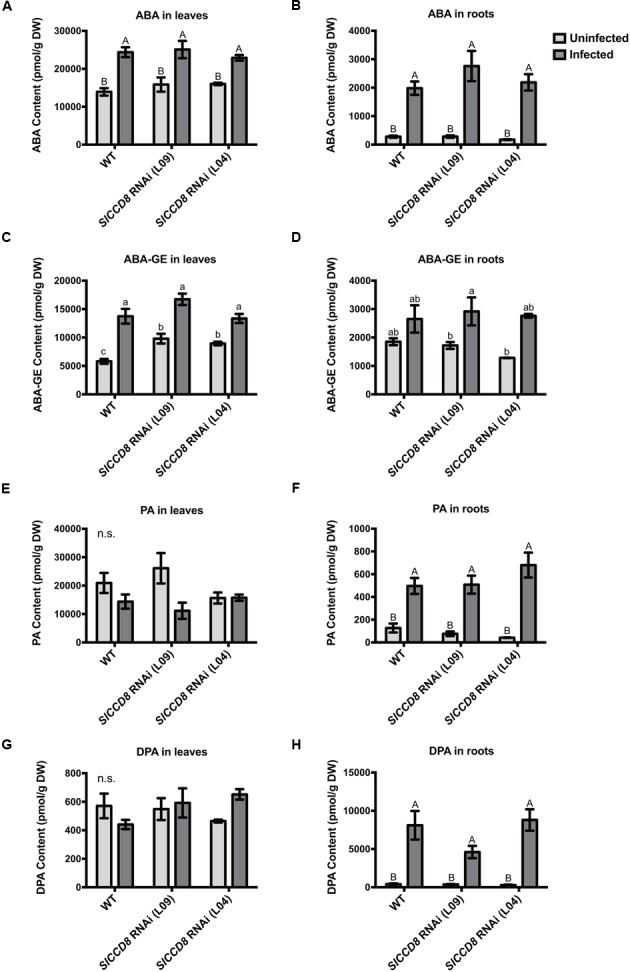
**Levels of abscisic acid (ABA) (A,B)** and ABA metabolites **(C–H)** in the leaves and roots of wild type (WT) and *SlCCD8* RNAi lines (L09 and L04) uninfected and infected with *P. ramosa*. ABA metabolites measured in this study include ABA conjugate ABA-glucose ester (ABA-GE) **(C,D)**, and two ABA degradation products phaseic acid (PA) **(E,F)** and dihydrophaseic acid (DPA) **(G,H)**. Data represent the means of five independent replicates ± standard error (SE). Letters (a, b, c and A, B) indicate statistically significant differences according to two-way ANOVA and Tukey’s multiple comparisons tests (a, b, c at the level of *P* < 0.05; A, B at the level of *P* < 0.01); n.s.: no significant differences for any of the comparisons in the respective graph.

As ABA has previously been shown to be involved in host–parasitic plant interactions (Taylor et al., 1996; Frost et al., 1997; Jiang et al., 2004, 2010), and ABA levels in the *SlCCD8* RNAi line have been reported to be decreased compared to wild type (Torres-Vera et al., 2014), levels of ABA and three derived metabolites, ABA-glucosyl ester (ABA-GE), phaseic acid (PA) and dihydrophaseic acid (DPA), were measured in roots and leaves of infected and uninfected wild type and *SlCCD8* RNAi lines in this study (**Figure 4**). Unexpectedly, uninfected *SlCCD8* RNAi lines had similar ABA levels in roots and shoots as the uninfected wild type plants (**Figures 4A,B**). In response to the *P. ramosa* infection, ABA levels in these tissues increased significantly to similar levels in all lines (**Figure 4A**, *P* < 0.01). As wild type and *SlCCD8* RNAi lines did not remarkably differ in ABA level in the leaves and roots when they were not infected, it can be concluded that the net increase in ABA was not different between wild type and *SlCCD8* RNAi lines. In contrast to ABA, the level of the major conjugate of ABA, ABA-GE, was higher in the leaves of uninfected *SlCCD8* RNAi lines (L09 and L04) than in leaves of uninfected wild type plants (**Figure 4C**, *P* < 0.05). Upon infection, ABA-GE levels of wild type plants and *SlCCD8* RNAi lines increased to a similar level (**Figure 4C**), suggesting that *P. ramosa* infection induced a higher net increase of ABA-GE in the WT plants. Uninfected wild type and *SlCCD8* RNAi lines had similar levels of ABA-GE in the roots (**Figure 4D**). Upon parasite infection, only one of the *SlCCD8* RNAi lines (L09) displayed a significant increase in ABA-GE level in the roots (**Figure 4D**), suggestive of a higher net increase of ABA-GE in the line L09 upon parasite infection. Regarding ABA catabolism, uninfected wild type and *SlCCD8* RNAi lines had similar levels of PA and DPA, in both leaves and roots, respectively (**Figures 4E,G**). Parasite infection strongly elevated the PA and DPA levels, but to a similar level in the roots of wild type and *SlCCD8* RNAi lines (**Figures 4F,H**).

**FIGURE 5:**
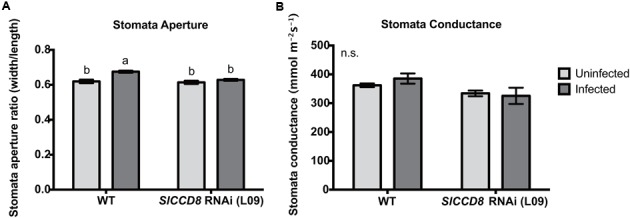
**Stomatal features of wild type (WT) and *SlCCD8* RNAi line L09 uninfected and infected with *P. ramosa.* Photos of stomata were taken of the leaf imprints and subjected to image analysis. (A)** Stomata aperture was measured by calculating the ratio of stomatal width to length. **(B)** Stomatal conductance (mmol m^-2^s^-1^) was measured directly on tomato leaves by using a leaf porometer in the afternoon. Data represent the means of 10 **(A)** and five **(B)** independent replicates ± standard error (SE). Letters (a, b) indicate statistically significant differences according to two-way ANOVA and Tukey’s multiple comparisons test (*P* < 0.05); n.s.: no significant differences for any of the comparisons in the respective graph.

**FIGURE 6:**
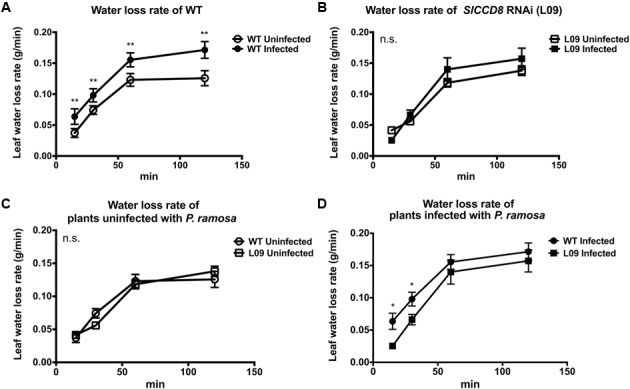
**Leaf water loss rate as observed in WT and *SlCCD8* RNAi line L09 uninfected and infected with *P. ramosa*.** Full-expanded leaves with similar age from the top of the plant were detached from tomato plants in the soil assay (8 weeks after infection). Collected leaves were placed in open Petri dishes for a dehydration assay. Leaf weights were periodically measured at the indicated time points (0, 15, 30, 60, 90 min). Rate of leaf water loss was calculated as leaf weight loss divided by the time interval. For clarity, comparisons of leaf water loss are shown separately between **(A)** infected and uninfected WT; **(B)** infected and uninfected L09; **(C)** uninfected WT and L09; **(D)** infected WT and L09. Data represent the means of four independent replicates ± standard error (SE). Asterisks (^∗^) indicate statistically significant differences based on Student’s *t*-test performed on each two cases in **(A,D)** at each time point. ^∗∗^*P* < 0.01; ^∗^*P* < 0.05); n.s.: no statistical significant differences for any of the comparisons in the respective graph.

ABA regulates stomatal behavior and as a consequence water fluxes in plants. Considering the above described observation that an infection with *P. ramosa* increases ABA levels in leaves of infected plants, we also evaluated stomatal aperture, stomatal conductance and leaf water loss rate as determined by a dehydration assay. The stomatal aperture, stomatal conductance and leaf water loss rate of wild type and L09 did not statistically differ when plants were not infected with *P. ramosa* (**Figures 5, 6C**). Also, when wild type and L09 were infected, their stomatal conductance did not differ nor change (**Figure 5B**). However, stomatal aperture was significantly increased by infection in wild type plants (*P* < 0.05), while it was unaffected in infected L09 (**Figure 5A**). When wild type and L09 were not infected with *P. ramosa*, water loss rate of their leaves did not differ (**Figure 6C**). However, when infected with the parasite, we observed a remarkable increase in leaf water loss rate in infected wild type plants (**Figure 6A**, *P* < 0.01), whereas there was no significant change in leaf water loss rate in the infected L09 plants (**Figure 6B**). During the early time points, infected wild type had a stronger leaf water loss than infected L09 (**Figure 6D**, *P* < 0.05).

## Discussion

Root parasitic weeds of the *Orobanchaceae* family are posing a great threat to crops but are difficult to manage. Current strategies to control these weeds are not effective largely due to the fact that a substantial part of their lifecycle occurs underground. Strategies to explore resistance mechanisms against these weeds are needed. Here, we studied the effect of strigolactones on the interaction between tomato and *P. ramosa*, with specific focus on the post-attachment process of the parasitic infection and the consequences on plant architecture and aspects of water loss.

Our results show that SL-deficient tomato lines display an enhanced infection and increased tubercle development rate upon inoculation with pre-germinated *P. ramosa* seeds. These results suggest that SLs play a positive role in the host defense against *P. ramosa* infection. Interestingly, it was recently reported that the expression of SL biosynthetic genes *SlD27* and *SlCCD8* is induced in *P. ramosa*-infected tomato roots (Torres-Vera et al., 2016). The induction of the expression *of SlD27* was stronger during the early stages of the infection, while the expression of *SlCCD8* increased over time (Torres-Vera et al., 2016). In addition, the transcription of the putative ortholog of the SL receptor, *D14*, in tomato *(SlD14)* was also induced during the late stage of the infection process (Torres-Vera et al., 2016). Combined with the results of the present study, this suggests that SL biosynthesis is triggered in the host plant upon infection and that SL signaling may play a role in the host defense against root parasitic plants.

One possible explanation for the high susceptibility of the *SlCCD8* RNAi lines to parasite infection that was observed in the present study, may be their enhanced auxin transport capacity and altered auxin levels, as was reported for the Arabidopsis SL-deficient mutant *max4* (Bennett et al., 2006). It was indeed shown that the tomato *SlCCD8* RNAi lines have increased adventitious root formation, probably resulting from higher auxin levels in the lower part of the stem (Kohlen et al., 2012). Interestingly, it was previously shown in Arabidopsis that polar auxin transport directs the xylem continuity between the host root and *P. aegyptiaca* tubercles (Bar-Nun et al., 2008). Perhaps the increased auxin transport capacity in *max4*, or in the present study in the *SlCCD8* RNAi lines, facilitates the formation of the vascular connection between host and parasite. A higher efficiency of this process could stimulate development and shorten emergence time of the parasite. An early emergence time of *S. hermonthica* in rice resulted in shorter rice plants and reduced plant weight and was therefore negatively correlated with parasitic plant tolerance (Kaewchumnong and Price, 2008). It is of interest that the major QTL for *Striga* tolerance in the latter study was later found to co-localize with the major QTL for SL levels (Cardoso et al., 2014). In both studies the same mapping population was used, and although the total number of emerged *Striga* shoots was higher on the high SL producing parent (germination was not standardized by pre-germination with GR24), the latter parent did appear to be more tolerant to an infection with *Striga*.

In addition to auxin, the recently described reduced levels of defense-related hormones JA and SA in the SL-deficient tomato *SlCCD8* RNAi lines (Torres-Vera et al., 2014) may also contribute to their increased susceptibility to parasite infection. Many studies have demonstrated the induction of expression of JA, SA and ethylene-dependent genes in the host (Arabidopsis, sunflower, tomato, Medicago) in response to an infection with *Orobanche/Phelipanche* spp. (Dos Santos et al., 2003b; Letousey et al., 2007; Dita et al., 2009; Torres-Vera et al., 2016). Further studies are still needed to reveal the possible links between SLs and (other) defense signaling pathways such as those involving JA, SA and ethylene. Moreover, these experiments should ideally be performed in a dynamic way, including different post-attachment stages, thus addressing the relative contribution of the various defense-related processes during different time windows of the infection process.

Consistent with previous reports on *Orobanche cernua* and *S. hermonthica* (Hibberd et al., 1998; Taylor and Seel, 1998), *P. ramosa* infection reduced the total biomass and plant height of its host (**Figures 2, 3**). However, unlike some other reports which showed a reduction of shoot biomass of the infected host (Barker et al., 1996; Mauromicale et al., 2008), total biomass of wild type plants in the present study was mainly reduced through a decrease in root biomass (**Figure 3**). This may be due to the different tomato cultivars that were used, and/or the different growing conditions. In our study, infected wild type plants displayed a more compact and dwarf-like shoot architecture, which was caused by a decrease in internode length (**Figures 2F,G**). Although, this phenotype resembled the SL-deficient lines to some extent, the numbers of primary and secondary branches did not increase in infected wild type plants (**Figures 2B,C**). Total biomass of the *SlCCD8* RNAi lines was also reduced upon infection (**Figure 3**). However, besides a large contribution of reduced root biomass (**Figure 3B**), the loss in biomass of *SlCCD8* RNAi lines was also due to a dramatic reduction in their initially higher number of secondary branches, as well as a reduction in the length of primary and secondary branches (**Figures 2B–E, 3A**). The strong reduction in branching in parasitized *SlCCD8* RNAi plants is likely associated with parasite-induced hormonal changes. An interesting hormone in this respect is ABA. In the present study, plant parasitism resulted in a major increase in root and shoot ABA levels of the host plant. Several reports have proposed a role for ABA in the inhibition of bud outgrowth (Suttle and Hultstrand, 1994; Emery et al., 1998; Shimizu-Sato and Mori, 2001; Suttle, 2004). Moreover, reduced ABA levels were observed in the lower buds of the high branching SL signaling mutant *max2*, while ABA application in this genotype resulted in partial suppression of branch elongation (Yao and Finlayson, 2015). This would place the axillary bud outgrowth inhibiting activity of ABA downstream of SL signaling and may explain the reduction in the number of secondary branches upon parasite infection in the SL-deficient *SlCCD8* RNAi tomato line observed in the present study.

Also in other studies, ABA has been considered to play a role in the interaction between the host and root parasitic plants. Increased expression of ABA biosynthetic genes and an abundance of ABA-responsive proteins were observed in tomato, pea and medicago parasitized by *P. ramosa* and *O. crenata* (Angeles Castillejo et al., 2004; Castillejo et al., 2009; Torres-Vera et al., 2016). It has been proposed that ABA biosynthesis in the host root might be triggered by local water deficiency around the haustoria (Taylor et al., 1996). In the present study, we observed that both root and shoot ABA levels in wild type and *SlCCD8* RNAi plants increased upon infection by the parasite to a similar extent. This ABA response can therefore not explain the observed difference in the *P. ramosa* infection level between the transgenic and WT lines. Also in uninfected plants, ABA levels in *SlCCD8* RNAi and wild type plants were similar which is in contrast to a previous study, where the SL-deficient line was described to have lower levels of ABA (Torres-Vera et al., 2016). However, in the present study, a higher level of the ABA conjugate, ABA-GE, was observed in the leaves of uninfected *SlCCD8* RNAi lines when compared with wild type, while the level of PA and DPA were similar. Cleavage of ABA-GE has been proposed as a rapid route for ABA production in response to drought and osmotic stress (Lee et al., 2006; Xu et al., 2012; Liu Z. et al., 2015). Drought and salt stress have been found to increase ABA-GE levels in the xylem in several cases (Sauter et al., 2002). Whether an increased conjugation rate of ABA in SL-deficient plants could contribute to their higher susceptibility to the parasite remains a question that needs further exploration.

Besides the increase in ABA levels in the host upon infection, ABA levels have also been reported to be increased in the parasitic plants themselves. For instance, *Orobanche* spp. (i.e., *Orobanche hederae*) accumulate high levels of ABA in their sink organs, i.e., inflorescence, which is in high demand for phloem-transported assimilates (Ihl et al., 1987). Reports on the interactions between hosts (maize and sorghum) and the hemiparasite *S. hermonthica* have shown that attached *Striga* plants accumulate much more ABA than their hosts, even though *Striga* infection also leads to increased ABA levels in the infected host (Taylor et al., 1996; Frost et al., 1997). Detailed modeling studies performed for the association between *R. minor* and barley suggested the formation of an ABA gradient between the parasite and host, which might contribute to an increased water flow from the host into the parasite (Jiang et al., 2004, 2010). Intriguingly, in the present study, the increase in ABA level in the host shoot upon parasite infection did not result in stomatal closure. On the contrary, in wild type tomato an increase in stomatal aperture was observed, resulting in an increased water loss rate. Interestingly, in parasitic plants such as *R. minor*, stomata remain open despite high ABA levels (Jiang et al., 2004). It has been suggested that the observed accumulation of high levels of cytokinin in leaves antagonizes ABA action, resulting in ABA insensitivity of the stomata (Jiang et al., 2005). It is not clear yet if this ABA insensitivity also occurs in *Orobanche*/*Phelipanche* species and whether it would influence ABA levels and/or ABA sensitivity in the host as well. It could be that in our study, the infected tomato plants also contain high levels of cytokinin, which might antagonize the effect of ABA on stomatal closure (Blackman and Davies, 1983; Tanaka et al., 2006), hereby preventing stomatal closure of the parasitized host. If so, it is of interest to point out that SLs also influence cytokinin levels. SL-deficient mutants have been reported to contain reduced levels of cytokinin in xylem sap (Beveridge et al., 1994, 1997a,b; Morris et al., 2001; Foo et al., 2007). Putative lower cytokinin levels in the SL-deficient *SlCCD8* RNAi tomato line that was used in the present study would explain why the stomatal aperture in infected *SlCCD8* RNAi plants was lower than in infected wild type plants, while ABA levels were similar in both genotypes.

## Conclusion

In the present study we have explored the effect of an infection with *P. ramosa* on host plant growth and architecture, its ABA and ABA metabolite profiles, stomatal conductance and water loss. Currently, there are only a few reports on the role of ABA during the interaction between the host and parasitic plants, and the role of ABA in the establishment of the water flow from host to parasite is unresolved. It is vital to study the dynamics of ABA and water flow and to build a proper model for the host–parasite association. It is also of interest to explore how the parasite prevents its host from closing its stomata regardless of the elevated ABA level in the host leaves. Intriguingly, our observations suggest that SL deficiency in tomato leads to an increased infection by parasitic plants which may have implications for future strategies on how to improve parasitic plant resistance. In this respect, the emphasis should be on the development of plants with reduced SL exudation rates or a low parasitic plant germination stimulating SL profile rather than on reducing SL content/production as a whole.

## Author Contributions

XC, HB, and CR-S designed the experiments. XC implemented most of the study, collected and analyzed the data. KF measured ABA levels and analyzed the hormonal data. XC and CR-S wrote the manuscript. XC, HB, and CR-S revised the manuscript.

## Conflictof Interest Statement

The authors declare that the research was conducted in the absence of any commercial or financial relationships that could be construed as a potential conflict of interest.
